# Adaptive Susceptible-Infectious-Removed Model for Continuous Estimation of the COVID-19 Infection Rate and Reproduction Number in the United States: Modeling Study

**DOI:** 10.2196/24389

**Published:** 2021-04-07

**Authors:** Mark B Shapiro, Fazle Karim, Guido Muscioni, Abel Saju Augustine

**Affiliations:** 1 Anthem, Inc Indianapolis, IN United States

**Keywords:** compartmental models, COVID-19, decision-making, estimate, infection rate, infectious disease, modeling, pandemic, prediction, reproduction number, SARS-CoV-2, United States

## Abstract

**Background:**

The dynamics of the COVID-19 pandemic vary owing to local population density and policy measures. During decision-making, policymakers consider an estimate of the effective reproduction number R_t_, which is the expected number of secondary infections spread by a single infected individual.

**Objective:**

We propose a simple method for estimating the time-varying infection rate and the R_t_.

**Methods:**

We used a sliding window approach with a Susceptible-Infectious-Removed (SIR) model. We estimated the infection rate from the reported cases over a 7-day window to obtain a continuous estimation of R_t_. A proposed adaptive SIR (aSIR) model was applied to analyze the data at the state and county levels.

**Results:**

The aSIR model showed an excellent fit for the number of reported COVID-19 cases, and the 1-day forecast mean absolute prediction error was <2.6% across all states. However, the 7-day forecast mean absolute prediction error approached 16.2% and strongly overestimated the number of cases when the R_t_ was rapidly decreasing. The maximal R_t_ displayed a wide range of 2.0 to 4.5 across all states, with the highest values for New York (4.4) and Michigan (4.5). We found that the aSIR model can rapidly adapt to an increase in the number of tests and an associated increase in the reported cases of infection. Our results also suggest that intensive testing may be an effective method of reducing R_t_.

**Conclusions:**

The aSIR model provides a simple and accurate computational tool for continuous R_t_ estimation and evaluation of the efficacy of mitigation measures.

## Introduction

The COVID-19 pandemic is currently underway. As of September 2, 2020, over 6,000,000 individuals in the United States have been reported positive for COVID-19. Modeling studies are key to understanding the factors that drive the spread of the disease and for developing mitigation strategies. Early modeling efforts forecasted very large numbers of infected individuals, which would overwhelm health care systems in many countries [1-3]. These forecasts served as a call to action for policymakers to introduce policy measures including social distancing, travel restrictions, and eventually lockdowns to avoid the predicted catastrophe [4-6]. The mitigating policy measures have been successful in changing the dynamics of the pandemic and in “flattening the curve,” such that fewer people have needed to seek treatment at any given time, and this has prevented the health care system from getting overwhelmed.

One of the most fundamental metrics that describes the pandemic’s dynamics is the reproduction number R_t_, which is the expected number of secondary infections spread by a single infectious individual [7]. In 1906, Hamer [8] speculated that the course of an epidemic is determined by the rate of contact between susceptible and infectious individuals. Later, Kermack and McKendrick [9] reported that epidemics end not when there are no susceptible individuals left, but rather when each infectious individual can infect, on average, <1 more individual. The R_t_ depends on three factors: (1) the likelihood of infection per contact, (2) the period during which infectious individuals freely interact with susceptible individuals and spread the disease, and (3) the rate of contact. The likelihood of infection per contact (factor 1) is determined on the basis of pathogen virulence and protective measures such as social distancing or wearing masks. Free interactions between infectious and susceptible individuals (factor 2) occur until the infectious individual is self-quarantined or hospitalized, either when the individual tests positive or experiences severe symptoms. Finally, the rate of contact (factor 3) is strongly affected by public health measures to mitigate risk [10], such as lockdowns during the COVID-19 pandemic. Thus, R_t_ is determined on the basis of the biological properties of the pathogen and multiple aspects of social behavior. When R_t_>1, the number of cases is expected to increase exponentially. The pandemic is considered to have been contained when R_t_ decreases and remains at <1. Real-time R_t_ estimation is critical for determining the effect of implemented mitigation measures and future planning.

We propose a method for continuous estimation of the infection rate and R_t_ to investigate the effect of mitigation measures and immunity acquired by those who recover from the disease. We estimated R_t_ with a Susceptible-Infectious-Removed (SIR) model [9] that describes the dynamics of population compartments as follows: individuals are initially “susceptible,” contract the viral infection and become “infectious,” and are then moved to the “removed” compartment once they are quarantined or hospitalized, recover, or die. The SIR model is one of the simplest epidemiological models that still captures the main properties of an epidemic [11,12], and it has been widely used in epidemic modeling studies. In most SIR modeling studies, the model parameters were constant. An SIR model with constant parameters, however, cannot be applied for the COVID-19 pandemic because various mitigating measures were introduced during pandemic progression. The effect of policy changes on COVID-19 dynamics has been modeled using the combination of an SIR model and Bayesian inference [13,14]. In these modeling studies, the rate of infection spread was assumed to be piece-wise linear among the 3 dates of the implementation of policy changes. In another approach, continuous estimation of R_t_ and an assessment of the effect of mitigation measures were carried out on the basis of estimates of the distribution of the serial intervals between symptom onset in the primary and secondary cases [15-17]. Bayesian inference and methods based on estimations of the serial interval include multiple parameters whose values are not estimated from the data. In contrast, we propose an adaptive SIR (aSIR) model in which only one parameter—the removal rate—is determined from the literature, while the second parameter—the infection rate—is continuously estimated from the data through a sliding window approach. A continuous R_t_ estimate is then obtained using the infection rate estimate. The SIR model is described as a system of differential equations, and the key idea in our proposed method is that the initial conditions for each window are considered as values estimated for the previous window. The only additional hyperparameter is the length of the sliding window. The proposed method retains the conceptual and computational simplicity of SIR-type models and can be easily extended through the introduction of additional compartments supported by data.

## Methods

### Data

Data on daily and cumulative confirmed cases between February 29 and September 2, 2020, were obtained from John Hopkins University (JHU), and the dates of interventions by state (eg, state of emergency and stay-at-home orders) were obtained from Wikipedia. The JHU data were available at 2 levels of aggregation: county and state. JHU considers many sources for reporting these data; county-level information was extracted from the websites of the states’ departments of health, and state-level data were extracted directly from the website of the Centers for Disease Control and Prevention.

### Model

The SIR model is a system of ordinary differential equations:


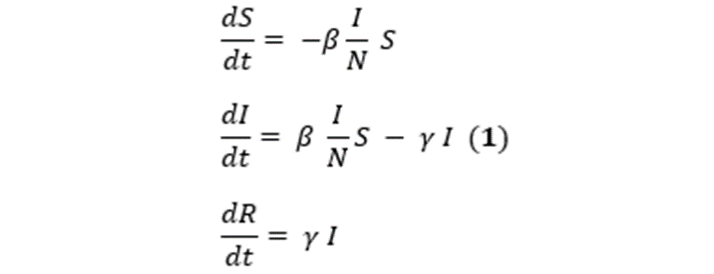


Here, *S* is the number of susceptible individuals, *I* is the number of infectious individuals who freely interact with others and can transmit the infection, *R* is the number of individuals excluded from the other 2 compartments because they are quarantined or hospitalized, have recovered and acquired immunity, or have died. Several sources of government data on COVID-19 provide the daily number of newly confirmed cases and a cumulative number of confirmed cases. Careful consideration is required to determine whether these numbers should be attributed to the *I* or *R* compartment. In the United States, once an individual has been confirmed positive for COVID-19, he/she is expected to be either self-isolated or hospitalized. Therefore, we assigned the data on confirmed cases to the *R* compartment, and we fit the model to the cumulative number of confirmed cases.

The infection rate is determined as follows:

*β* = *p* × *c*           **(2)**

where *p* is the probability of being infected upon contact with an infectious individual, and *c* is the average number of contacts per day. We have no data that would allow us to estimate *p* and *c* separately; hence, we directly estimated *β*, as is usually performed when using SIR models.

The removal rate *γ* determines the rate at which infected individuals are moved from the *I* to the *R* compartments. In the context of the COVID-19 pandemic, *γ* is determined from the time taken for the appearance of severe symptoms, such that the individual can be tested and is self-quarantined or hospitalized, as required. Therefore, we assumed the duration of the infectious period as the average time taken for the infected individual to be isolated, not the overall time for recovery. We assumed that an individual is infectious from the day he/she contracts the infection before symptom onset [18-20]. The average time to symptom onset is 5-6 days [21-23]. We assumed that the infectious period before the development of severe symptoms is 6 days; hence, *γ*=1/6.

### Time-Variant Parameter Estimation

The aSIR model contains two parameters, *β* and *γ*, with *γ*=1/6 obtained from the literature, and *β* estimated from the reported data for each region of interest. The time-variant *β*(t) was estimated using a sliding window of τ=7 days and step of *s*=1 day, with the estimated values for *S* and *I* obtained from the previous window used as the initial conditions for the next window.

The reproduction number was calculated as follows:

*R_t_*(t) = *β*(t)/*γ*           **(3)**

For the first window, we determined the date when the number of confirmed cases began to increase exponentially. This is important because for many states or counties, very few confirmed cases were initially reported for a number of days or even weeks, which suggests that either the epidemic had not started or the true number of infected individuals was unknown. It is not reasonable to apply an SIR model for this initial period. We considered the onset of the pandemic as the first of the 4 consecutive days in which the number of reported confirmed cases increased in at least 3 days. The initial conditions for system (1) for window 0 were as follows:

*S_0_(0)* = *N*           **(4)**

where *N* is the population in the region of interest, *I_0_(0)*=1, and *R_0_(0)*=0. The infection rate β*_i_* and *S(t), I(t)* for *t* [0, *τ—1*] were estimated from the initial conditions and actual *R*.

The window was slid by *s*=1 point. For the new *i+1* window, the initial conditions were considered as the estimated values from the previous window *S_i+1_(0)*=*S_i_(s), I_i+1_(0)*=*I_i_(s),* and actual *R_i+1_(0)*=*R(s)*. The actual values of *R(t)* were used*,* and the infection rates *β_i+1_* and *S_i+1_(t)*, and *I_i+1_(t)* were estimated*.*

For each window, the *R_t.i_* was determined as follows:

*R_t.i_* = *β*_i_/*γ*           **(5)**

The *R_t.i_* was assigned to the last time point of the window. To obtain a smooth estimate of R_t_, we used a rolling average of 5 points.

## Results

**Figure 1:**
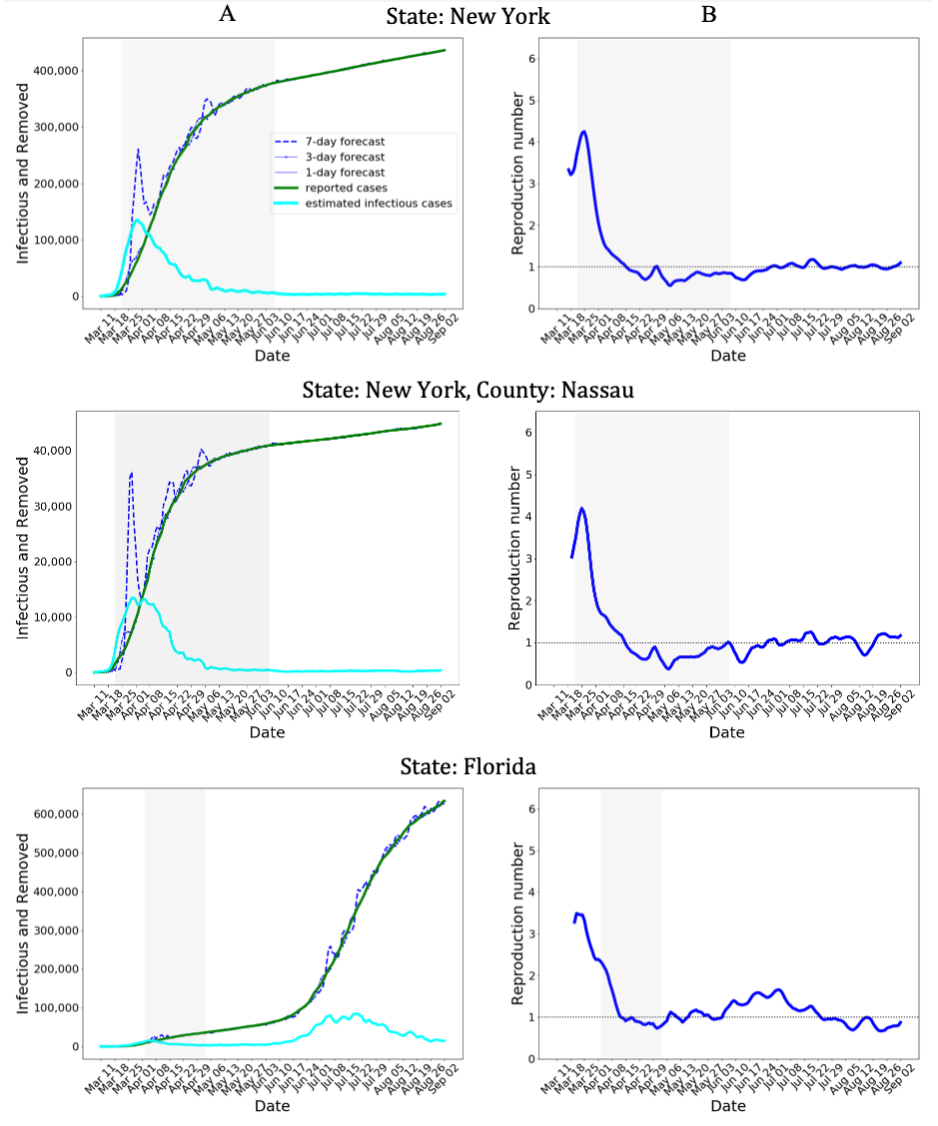
(A) Estimated Infectious and forecast Removed. (B) Estimated reproduction number R_t_. The shaded region indicates the dates of the lockdown. While the 1-day and 3-day forecasts are accurate, the 7-day forecast exhibits marked errors when R_t_>1 and is rapidly decreasing.

We fit the model for each state and county in the United States. Model performance was evaluated by calculating the quality of fit as the root mean squared error between the actual and fitted *R* data for all windows concatenated (wRMSE). The fit was excellent with wRMSE<6 across all states. Furthermore, we calculated 1-day, 3-day, and 7-day forecasts of *R* after each window (Figure 1A). The mean absolute prediction error for the forecasts is provided in Table 1. The 1-day forecast error did not exceed 2.6% across all states, while the 7-day forecast error was large and approached 16.2% for New York. In particular, the 7-day forecast strongly overestimated the number of cases when R_t_ was rapidly decreasing (Figure 1).

**Table 1:** Reproduction numbers and forecast accuracy for 50 US states.

State	R_t_^a^ max	MAPE^b^ (1-day forecast), %	MAPE (3-day forecast), %	MAPE (7-day forecast), %
Alabama	2.9	1.5	4.2	10.0
Alaska	2.8	1.6	3.7	11.3
Arizona	3.3	1.3	2.9	10.1
Arkansas	2.8	1.5	4.0	12.6
California	2.5	1.7	2.9	6.3
Colorado	2.6	1.1	2.9	7.3
Connecticut	4.1	2.0	3.1	9.3
Delaware	2.4	1.7	2.9	7.9
District of Columbia	2.1	0.8	1.8	4.4
Florida	3.6	2.0	4.4	9.3
Georgia	3.0	1.8	3.7	7.4
Hawaii	2.7	2.0	3.6	9.7
Idaho	3.4	2.4	4.8	13.6
Illinois	4.0	1.3	2.5	8.5
Indiana	3.8	1.4	4.0	10.4
Iowa	2.8	1.8	3.6	8.0
Kansas	3.0	1.6	3.5	8.6
Kentucky	3.0	2.6	4.9	11.2
Louisiana	3.7	1.8	4.0	12.1
Maine	2.0	1.2	2.8	6.7
Maryland	3.3	1.2	2.8	6.2
Massachusetts	3.4	1.3	3.6	9.7
Michigan	4.5	1.6	3.5	12.8
Minnesota	2.7	1.3	2.9	8.0
Mississippi	2.9	1.2	3.0	9.3
Missouri	3.6	1.8	3.3	11.4
Montana	3.3	1.6	3.7	11.9
Nebraska	2.5	2.0	4.1	9.7
Nevada	2.9	2.3	3.8	10.0
New Hampshire	2.3	1.7	3.3	8.5
New Jersey	4.1	1.5	2.3	7.8
New Mexico	2.3	2.2	3.4	7.3
New York	4.4	1.5	4.2	16.2
North Carolina	3.2	1.3	2.4	7.2
North Dakota	2.4	1.8	4.8	12.6
Ohio	3.3	1.2	3.4	9.8
Oklahoma	3.1	1.4	3.5	10.2
Oregon	2.5	1.3	2.8	6.6
Pennsylvania	3.2	1.7	2.9	6.3
Rhode Island	2.4	1.5	3.1	6.8
South Carolina	3.5	2.1	4.3	10.6
South Dakota	2.1	1.3	3.2	8.7
Tennessee	3.5	2.2	4.8	12.5
Texas	3.6	2.0	3.9	9.3
Utah	3.2	1.4	3.1	8.3
Vermont	2.9	0.8	2.4	7.7
Virginia	2.5	1.1	2.1	5.1
Washington	3.0	2.0	4.8	8.6
West Virginia	3.5	1.6	3.9	14.0
Wisconsin	3.6	1.5	3.2	10.0
Wyoming	2.9	1.9	4.7	14.2

^a^R_t_: reproduction number.

^b^MAPE: mean absolute prediction error.

The estimated time course of R_t_ for New York and Nassau county, one of the most affected counties since the beginning of the COVID-19 pandemic, are shown in Figure 1. The estimated daily number of infectious individuals rapidly increased and then gradually declined after the lockdown was implemented on March 22, 2020 (Figure 1A). The estimated R_t_ also declined upon implementation of the lockdown (Figure 1B). The time course of R_t_ exhibits weekly seasonality, which likely reflects the effect of social interactions and possibly the effect of fluctuations in case reporting on weekdays vs weekends. For New York and Nassau county, R_t_ initially increased, which may reflect the fact that the pandemic in New York was continuously seeded by travelers arriving at John F Kennedy International Airport until a ban on international travel was implemented on March 12, 2020. This may also reflect the fact that not all severe cases were initially recognized and reported as COVID-19 cases. In Florida, R_t_ decreased to almost 1 by mid-April but then began increasing at the end of May (Figure 1B). In June 2020, Florida authorities introduced more stringent measures to control the pandemic, which is reflected in the reduction in R_t_ in the second half of July 2020. The opening of multiple states since June 2020 has been accompanied by an increase in R_t_ beyond 1 (data not shown), and close monitoring of R_t_ is needed to contain another wave of the pandemic.

**Figure 2:**
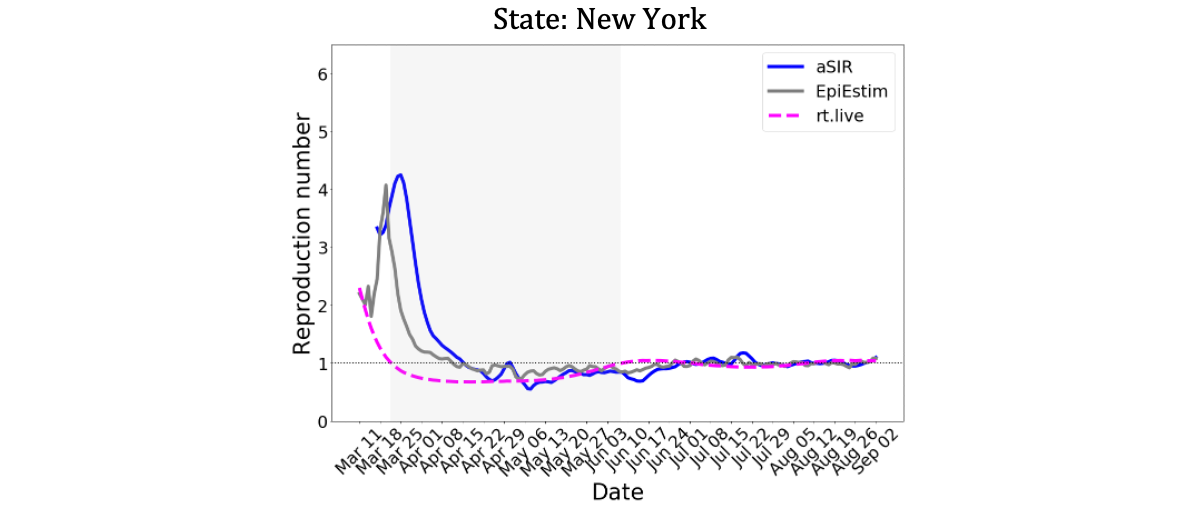
Comparison of models that generate continuous R_t_ estimates. The three R_t_ estimates differ widely in the beginning of the COVID-19 pandemic. In particular, the R_t_ estimated using the rt.live model of Systrom, Vladeck, and Krieger [24] decreased to 1 at the onset at the lockdown on March 22, 2020 (shaded region). aSIR: adaptive Susceptible-Infectious-Removed.

Next, we compared aSIR with the model developed by Cori et al [15], implemented as R package EpiEstim, and a model implemented by Systrom, Vladeck, and Krieger in rt.live [24] (Figure 2). In EpiEstim, we assumed an equal probability of infection within the infectious period of 6 days, the R_t_ estimate was smoothed with a 7-point rolling average window, same as that in aSIR. While all 3 models show similar estimates when R_t_ approaches 1, their estimates differ considerably in the beginning of the pandemic. In particular, the rt.live model [24] returned a lower maximum R_t_ than the other 2 models and estimated that R_t_ already decreased to 1 by the time the lockdown was announced in New York on March 22, 2020 (Figure 2, shaded region). The EpiEstim and aSIR models estimated similar peak values of R_t_, and both models estimated that R_t_ decreased and approached 1 in the first week of April 2020. Although both models show a rapid reduction in R_t_ in March, the aSIR model shows a lagged change. However, we are not aware of the ground truth data to determine which model yields a more accurate estimate.

**Figure 3:**
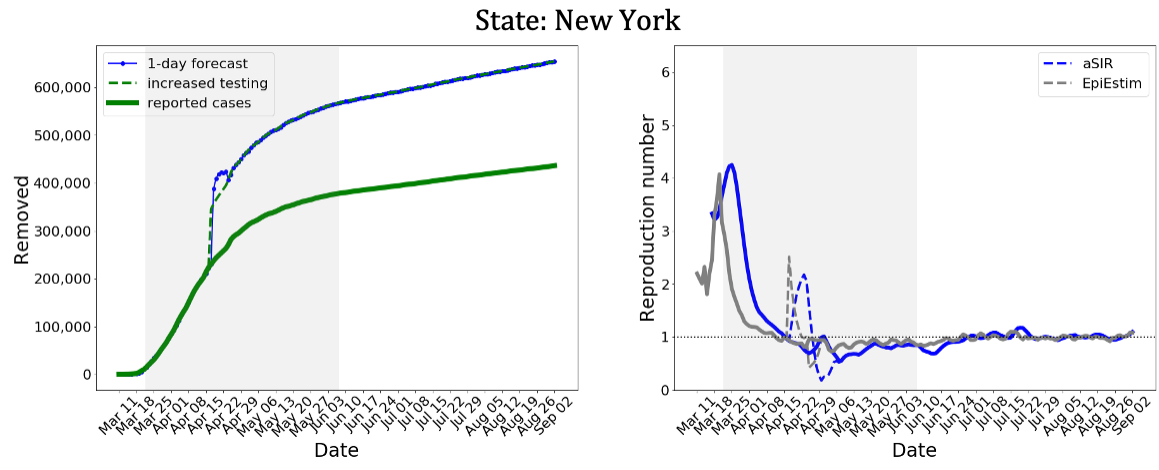
Effect of a step-wise 50% increase in testing (left panel, dashed line). The 1-day forecast by the aSIR model adapts within a week. For the Rt estimate, both EpiEstim and our aSIR models produced a spike, followed by a reduction (right panel, dashed lines) before returning to the unperturbed Rt time course (solid lines). aSIR: adaptive Susceptible-Infectious-Removed.

Finally, we investigated the effect of an abrupt increase in testing on the estimated R_t_ (Figure 3). We assumed a step-wise 50% increase in testing, which persisted after April 12, 2020 (Figure 3, left panel). Both aSIR and EpiEstim models exhibited a spike in R_t_. However, an increase in testing would help identify and quarantine infectious individuals sooner, resulting in a shorter infectious period and larger removal rate *γ*, in turn decreasing R_t_. We did not model a potential increase in *γ*. Instead, we assumed that the underlying dynamics of the pandemic did not change, and within 2 weeks both models returned to the R_t_ time course estimated without an increase in testing.

## Discussion

### Principal Findings

We developed a simple approach to adaptively estimate the time-varying parameters of the SIR model, using reported data on the number of confirmed COVID-19 cases. This approach adds to the already large literature on COVID-19 modeling in 2 ways. First, we estimate the parameters of the SIR model with a sliding window of a limited duration (7 days) to account for rapid changes in transmissibility and contact patterns in response to changes in social behavior and government mitigation measures. The window duration is a hyperparameter that can be changed as needed, the trade-off being the accuracy of the parameter estimates versus the rapid reaction to changes in the underlying pandemic. Because the proposed model is so simple, a number of scenarios can be explored as needed.

Second, we attribute the data on reported cases to the Removed compartment rather than the Infectious compartment. This modeling decision is based on the realities of the COVID-19 pandemic in the United States, where individuals with confirmed COVID-19 are supposed to self-isolate or be hospitalized. Although these individuals remain infectious and can infect other family members or caregivers even when self-isolated or hospitalized, they would not freely interact with the susceptible population, as would be required to attribute them to the *I* compartment. The addition of a new X compartment in the SIR model has been proposed to model symptomatic quarantined infectious individuals [25]. However, we have no data to independently estimate this additional parameter of quarantine rate. For the same reason, we did not use the Susceptible-Exposed-Infected-Removed (SEIR) model because we are not aware of reliable data on the duration of the exposure period during which an infected person is not yet infectious. Moreover, it has been reported that the SIR model performed better than the SEIR model in representing the information contained in the confirmed-case data on COVID-19 [26].

The reported number of positive COVID-19 cases represents a fraction of infected individuals because of the limited testing capacity in March and April 2020; consequently, only those who developed severe symptoms were tested. Up to 80% of infected individuals may have been asymptomatic or may have experienced mild symptoms [27] and were not tested; hence, for that period, our model applies only to the small subpopulation with severe symptoms. However, this subpopulation is of particular interest because it represents those who are at the greatest risk, and R_t_ estimated from these limited data can be used to guide policy decisions aimed at protecting the most vulnerable population [28]. As the number of the tested individuals increases, the short sliding window approach makes our model adaptable to an increasing proportion of the population (Figure 3).

Across all US states, the maximal R_t_ values were estimated for New York (4.4) and Michigan (4.5) (Table 1), which is similar to the mean value of 4.34 estimated for Italy [29] but higher than that obtained with a stochastic transmission model [30,31]. The wide range of maximal values of R_t_ of 2.0-4.5 (Table 1) likely reflects the differences in contact rates owing to the population density [32,33]. Increased social distancing is required to contain the spread of the pandemic [34,35], with more stringent mitigation measures, including lockdown, considered necessary to decrease the contact rate in high-density states and counties. Another measure to decrease R_t_ is to increase the removal rate *γ* through intensive testing and quarantining of individuals who test positive. This targeted intervention would strongly decrease the interaction between infectious and susceptible individuals and maintain an R_t_ of <1 until a vaccine is available and while vaccination efforts are ramping up. Intensive testing combined with social distancing and mask wearing, followed by the isolation of individuals confirmed with COVID-19, are key features of reopening strategies for schools and universities [36-38]. Our model allows researchers and policymakers to monitor R_t_ in different geographic regions of the United States, better understand the effect of government policies on the dynamics of the pandemic, and develop further mitigation strategies as we continue to battle COVID-19 [39,40].

### Limitations

The SIR model is perhaps the simplest model that captures the dynamics of a pandemic. It is based on several assumptions that are valid only to some degree as we consider real-life scenarios. The 2 main limitations of the original SIR model are that it has constant parameters and it is deterministic. Our proposed aSIR model allows us to estimate time-varying parameters and thus removes the first limitation. The other limitation remains, however. It is assumed that infectious individuals freely interact with the susceptible population. The infection rate *β* encompasses both the probability of transmission and the average number of contacts per day. The SIR model does not reflect interaction dynamics that are stochastic in nature and are described by stochastic epidemiologic models [15-17,41]. In its simplest form, the SIR model does not reflect the heterogeneity of viral transmission reflected in overdispersion or superspreading where few numbers of infected individuals infect a large number of susceptible individuals [42-44]. The removal rate *γ* is an average number of days until an infectious individual is excluded and does not reflect the variability of this interval, nor does it allow one to model a possibility of a subsequent, albeit reduced transmission to caregivers or other susceptible individuals as may happen in a real-life scenario. Consequently, R_t_, which is calculated using constants *β* and *γ* rather than their distributions, does not reflect the stochastic nature of the dynamics of the pandemic. Parameter distributions can be obtained by applying Bayesian methods to SIR modeling [45]. Moreover, a single value of R_t_ estimated for a large population does not reflect differences in subpopulations, such as age groups, which is especially relevant for COVID-19 [46-48]. The generation of a bank of aSIR models for each subpopulation or region can provide a more realistic insight into the dynamics of the pandemic across a larger population [49,50]. Another critical assumption is that once an individual is infected and recovers, he/she is no longer susceptible to repeated infection; however, this assumption does not appear strongly violated. Although some cases of repeated infection with SARS-CoV-2 have been reported [51-53], the risk of re-infection is considered low [54,55]. Overall, the SIR model is a trade-off between the computational simplicity and veracity of describing the real-life complexities of a pandemic.

### Conclusions

SIR type models, particularly the proposed time-variant aSIR model, have an advantage over more complex models in the initial stages of a pandemic when critical public policy decisions need to be made while the empirical data on interaction dynamics, transmission rates, and the disease progression and contagiousness from the moment of infection are not yet readily available. Our model provides a simple and efficient method to assess the efficacy of interventions as the pandemic progresses.

## Abbreviations

SIR: Susceptible-Infectious-Removed
aSIR: adaptive Susceptible-Infectious-Removed
JHU: Johns Hopkins University
